# Treatment of Recurrent Hepatitis C Genotype-4 Post-Liver Transplantation with Sofosbuvir plus Simeprevir

**Published:** 2015-05-01

**Authors:** Mustafa Ascha, Mona Ascha, N. N. Zein, N. Alkhouri, B. Eghtesad, K. Abu-Elmagd, T. Diago, I. A. Hanouneh

**Affiliations:** 1 Department of Gastroenterology and Hepatology, Cleveland Clinic, Cleveland, Ohio, United States; 2 *Department of General Surgery, Transplant Center, Cleveland Clinic, Cleveland, Ohio, United States*

**Keywords:** Hepacivirus, Liver transplantation, Genotype, Interferon

## Abstract

Hepatitis C virus (HCV) infection remains a leading indication for orthotopic liver transplantation (OLT) worldwide. Recurrence of HCV following OLT is universal. There is scarcity of data on the post-OLT treatment of HCV genotype-4—the predominant genotype in North Africa and the Middle East. Herein, we present three patients who have experienced HCV genotype-4 recurrence post-OLT. All three patients were interferon-naive and were treated with simeprivir (SIM) and sofosbuvir (SOF) combination therapy for 12–24 weeks. The data from this case series show that SIM+SOF are well-tolerated and effective for achieving viral clearance in HCV genotype-4 post-OLT patients. Given the limited nature of a case series, further research must be pursued regarding post-OLT HCV genotype-4 responses to direct-acting anti-viral therapy.

## INTRODUCTION

While hepatitis C virus (HCV)-related cirrhosis remains the leading cause of liver transplantation, the majority of transplant patients experience re-infection with HCV as well as faster disease progression [1]. Post-transplantation HCV has been challenging to manage, as many treatment factors (*eg*, optimal immunosuppressive therapy, and anti-viral therapies) are highly complex. It is suggested that immunosuppression coupled with episodes of graft rejection increase the risk of HCV progression post-transplant [1]. While a variety of treatment regimens for patients who have been re-infected with HCV post-transplant are still being explored, little is known about the outcome of treatment for HCV genotype-4. The purpose of this case series was to present a promising 12–24-week treatment regimen for post-transplant HCV genotype-4 infection that involves two direct-acting anti-viral agents—sofosbuvir and simeprevir.

Among other patient characteristics, HCV genotype has been an important clinical factor in determining treatment response. There are at least six major HCV genotypes, each with multiple subtypes and quasispecies [2]. Genotype-4, the focus of this paper, is prevalent in North Africa and the Middle East. Egypt has the highest HCV prevalence in the world at 14.7%, and more than 90% of cases are genotype-4 [3]. Previous reports have shown that treatment with conventional interferon therapy is effective in patients with HCV genotype-4, but with only 40%–50% response rate [4, 5]. Furthermore, the treatment with interferon is poorly tolerated and is associated with significant adverse events [6].

In the last few years, there has been experimentation with therapies involving direct-acting anti-viral therapies, such as sofosbuvir and simeprevir. Last year, sofosbuvir became approved for use in triple therapy along with pegylated interferon and ribavirin for HCV genotype-4 [7]. Sofosbuvir is an oral nucleotide analogue inhibitor of the HCV-specific NS5B polymerase that has demonstrated improved patient outcomes after shorter treatments [8]. In addition, simeprevir is an HCV protease inhibitor that inhibits viral maturation. It has also been used with pegylated interferon and ribavirin as a triple therapy for HCV genotype-4 [9]. More recent research has focused on providing interferon-free therapy for HCV patients, to avoid debilitating side effects and to shorten treatment. It is well known that interferon treatment is very poorly tolerated by patients, thus research into alternative therapy is pivotal [8]. Direct-acting anti-viral agents, such as sofosbuvir and simeprevir, along with other agents currently being developed, have shown much promise in this new era of HCV treatment.

In this case series, we present three patients who have experienced HCV genotype-4 recurrence post-liver transplant. All three patients were treated with combination therapy of sofosbuvir and simeprevir that successfully eradicated the virus, demonstrating the efficacy of this treatment regimen for post-transplant genotype-4 HCV reinfection (Table 1). 

**Table 1:** Laboratory values in post-transplant HCV genotype-4 patients before and after sofosbuvir and simeprivir combination therapy

	Lab test	Before treatment	At the end of treatment	12 weeks after treatment
Patient 1	AST (U/L)	64	23	29
	ALT (U/L)	43	16	18
	Alkaline phosphatase (U/L)	172	70	75
	Total bilirubin (mg/dL)	2.1	1.2	1.1
	Hemoglobin (g/dL)	13.1	14.0	13.5
	HCV RNA (IU/mL)	3,230,000	Undetectable	Undetectable
Patient 2	AST (U/L)	53	18	20
	ALT (U/L)	59	22	35
	Alkaline phosphatase (U/L)	161	92	86
	Total bilirubin (mg/dL)	0.9	0.7	0.8
	Hemoglobin (g/dL)	12.9	13.2	13.0
	HCV RNA (IU/mL)	6,269,000	Undetectable	Undetectable
Patient 3	AST (U/L)	49	26	26
	ALT (U/L)	63	23	31
	Alkaline phosphatase (U/L)	159	102	79
	Total bilirubin (mg/dL)	1.0	0.9	0.9
	Hemoglobin (g/dL)	14.1	13.5	13.9
	HCV RNA (IU/mL)	2,586,000	Undetectable	Undetectable

**CASE 1**

The first case was a 62-year-old Caucasian man who received an orthotopic liver transplant (OLT) in 2005, indicated by cirrhosis due to alcohol abuse and HCV of genotypes 1 and 4. Post-transplant, the patient had done well with no episodes of acute cellular rejection and no other major complications. The patient was of a healthy BMI of 26 kg/m^2^, with no history of smoking but controlled diabetes. Past medical history is otherwise unremarkable.

Long-term post-transplant immunosuppressive regimen was 0.5 mg tacrolimus monotherapy twice daily. The patient was interferon-naïve. Last liver biopsy performed seven years post-OLT showed histological changes consistent with recurrent HCV with Batts and Ludwig grade 2/4 and stage 4/4 fibrosis. Patient has no ascites or hepatic encephalopathy. Recent upper endoscopy showed small non-bleeding esophageal varices. Liver function tests (LFTs) are presented in Table 1.

The patient agreed to initiate HCV treatment with a combination of sofosbuvir 400 mg daily and simeprevir 150 mg daily for 24 weeks due to the presence of cirrhosis. Immunosuppression treatment was not altered during HCV therapy. The patient did not encounter any side effects from the treatment. At the conclusion of therapy, the patient exhibited sustained viral clearance (SVR) with tests negative for HCV RNA at week 12 following completion of therapy. LFTs all normalized by the end of the treatment regimen. 

**CASE 2**

The second case was a 55-year-old Egyptian man who received OLT in 2010, indicated by HCV and hepatocellular carcinoma. The patient had recurrence of chronic HCV genotype-4. Post-transplant, the patient had done well, surveillance abdominal imaging was negative for recurrence of hepatocellular carcinoma. He had normal renal function, and normal LFTs. The patient was of a healthy BMI of 25 kg/m^2^, with a history of well-controlled diabetes and hypertension. Past medical history was also significant for smoking, schistosomiasis, hyperlipidemia and coronary artery disease.

Long-term post-transplant immunosuppressive regimen was 1 mg tacrolimus monotherapy twice daily per oral with tacrolimus levels maintained from 3–5 ng/mL. The patient was interferon-naïve. Recent liver biopsy showed recurrent HCV, Batts and Ludwig grade 2/4 with stage 0/4 fibrosis. 

The patient agreed to initiate HCV treatment with a combination of sofosbuvir and simeprevir for 12 weeks. At a pre-therapy appointment, patient was anicteric with no adenopathy; the abdomen was soft with no ascites, mass or organomegaly; and there was no edema. AST and ALT levels prior to the treatment were mildly elevated (Table 1). LFTs normalized following initiation and at the end of the treatment. At the conclusion of therapy, the patient exhibited SVR with tests negative for HCV RNA at week 12 following completion of anti-viral therapy. The patient did not encounter any side effects from the treatment. Immunosuppression treatment was not altered during HCV therapy.

**CASE 3**

The third case was a 59-year-old Egyptian man who received an OLT in 2012, indicated by cirrhosis due to chronic HCV genotype-4 infection. At the time of transplant, there was an incidental finding of a single 0.9-cm lesion of hepatocellular carcinoma. Post-transplant, the patient had recurrent HCV infection. The patient was overweight at a BMI of 29 kg/m^2^. His past medical history was notable for childhood schistosomiasis. The patient quit smoking in 1980 and did not drink alcohol or use recreational drugs. Post-transplant course had been complicated by metabolic risk factors including hypertension, uncontrolled diabetes mellitus, hyperlipidemia, and stage-3 chronic kidney disease. 

Long-term post-transplant immunosuppressive regimen included 1.5 mg tacrolimus twice daily per oral with tacrolimus levels maintained at 4.3 ng/mL. The patient was interferon-naïve. Last liver biopsy in 2013 showed patchy, mild, nonspecific chronic inflammation in the portal region. Trichrome stain showed no evidence of fibrosis. Batts and Ludwig grade was 1/4 with stage 0/4 fibrosis. 

A 12-week regimen of sofosbuvir and simeprevir was initiated. At first the patient had no edema, was anicteric and in no apparent distress. The patient had mildly elevated AST and ALT; the remaining LFTs were otherwise normal (Table 1). LFTs all normalized following initiation and at the end of the treatment. At the conclusion of therapy, the patient exhibited SVR with tests negative for HCV RNA at week 12 following completion of anti-viral therapy. The patient did not encounter any side effects from treatment. Immunosuppression treatment was not altered during HCV therapy.

## DISCUSSION

Our experience showed that sofosbuvir plus simeprevir are well-tolerated and effective for viral clearance in patients who have HCV genotype-4 post-OLT. The last few years have been an exciting time for HCV treatment. New treatment options involving combinations of direct-acting anti-viral agents have provided promise for developing more effective and less burdensome therapy. In particular, sofosbuvir is exceptional because of its high potency and high genetic barrier to resistance [10]. When sofosbuvir was used in combination with other direct-acting anti-viral agents, treatment outcomes were encouraging, demonstrating high SVR rates [11].

It is important to understand how specific populations will respond to a new therapy. Given the current context of novel HCV therapy, our study examined treatment in a specific population defined by two important characteristics—post-OLT HCV and HCV genotype-4. 

Immunosuppressive therapy in post-transplant patients paves the way for new infections to thrive or previous infections to recur [11]. Post-OLT HCV treatment is not standardized reflecting the fact that existing literature on the topic is inconclusive. Success in treating patients with combination simeprevir and sofosbuvir can contribute to the growing understanding post-OLT HCV treatment. 

HCV genotype-4 has been a challenge for physicians to treat. The majority of published data are focused on HCV genotype-1 treatment, because it is the most common genotype in the USA. However, it is important to develop an understanding of optimal treatment for HCV genotype-4, as it is highly endemic in other parts of the world, particularly in North Africa. Knowledge of optimal treatment for HCV genotype-4 is valuable because it is the principal subtype in geographic areas where chronic hepatitis C is highly prevalent. On a global scale, those infected with HCV genotype-4 represent a large proportion of patients with chronic hepatitis C [6]. HCV genotype-4 is also becoming increasingly common in Western countries, particularly in Europe [12]. In the USA, physicians who provide care for immigrants from corresponding endemic areas must have knowledge of the latest therapies for HCV genotype-4 [13]. Furthermore, direct-acting anti-viral agents (*eg*, sofosbuvir) have very recently become available in endemic areas such as India and Egypt. Therefore, a comprehension of the efficacy of these drugs is critical. The USA is also a center for pharmaceutical innovation, and the potential consequences of its drug and therapeutic developments must be considered in a global context. Information regarding HCV genotype-4 treatment post-transplant is limited [14, 15]. This study can contribute to growing understanding of the second generation direct-acting anti-viral therapies for HCV genotype-4, particularly post-transplant. 

This study was limited in there is no information on long-term follow-up of the cases. Given the novelty of the second generation direct-acting anti-viral therapies, there must be adequate long-term follow-up for all patients treated with these drugs. In addition, this study was also limited in that direct-acting anti-viral agents are very expensive drugs. The high cost for these drugs may significantly hinder their accessibility, particularly within populations where they are most needed. However, some pharmaceutical corporations are addressing this need by selling drugs at less expensive prices in developing nations such as Egypt and India [16]. This may enhance the availability of the drug in endemic areas.

Continued research of HCV genotype-4 responses to new antiviral agents is vital. Future prospective studies of treatment for HCV genotype-4 recurrence post-OLT should involve larger cohorts. Published data regarding HCV recurrence post-OLT and treatment for HCV genotype-4 are discordant, reflecting the fact that there is an unclear understanding of these topics. In the interest of maximizing organ donation outcome, post-transplant responses to treatment must be examined well. This study serves as preliminary data for research evaluating HCV treatment post-OLT and HCV genotype-4 treatment. 
